# Cytokine-induced killer cells promote antitumor immunity

**DOI:** 10.1186/1479-5876-11-83

**Published:** 2013-03-28

**Authors:** Jingting Jiang, Changping Wu, Binfeng Lu

**Affiliations:** 1 Department of Tumor Biological treatment, the Third Affiliated Hospital of Soochow University, 185 Juqian Street, Changzhou, 213003, China; 2 Department of Immunology, University of Pittsburgh School of Medicine, 200 Lothrop Street E1047, Pittsburgh, PA, 15261, USA

## Abstract

The number of immune cells, especially dendritic cells and cytotoxic tumor infiltrating lymphocytes (TIL), particularly Th1 cells, CD8 T cells, and NK cells is associated with increased survival of cancer patients. Such antitumor cellular immune responses can be greatly enhanced by adoptive transfer of activated type 1 lymphocytes. Recently, adoptive cell therapy based on infusion of *ex vivo* expanded TILs has achieved substantial clinical success. Cytokine-induced killer (CIK) cells are a heterogeneous population of effector CD8 T cells with diverse TCR specificities, possessing non-MHC-restricted cytolytic activities against tumor cells. Preclinical studies of CIK cells in murine tumor models demonstrate significant antitumor effects against a number of hematopoietic and solid tumors. Clinical studies have confirmed benefit and safety of CIK cell-based therapy for patients with comparable malignancies. Enhancing the potency and specificity of CIK therapy via immunological and genetic engineering approaches and identifying robust biomarkers of response will significantly improve this therapy.

## Introduction

The presence of cytotoxic tumor infiltrating lymphocytes (TIL) within tumor is associated with increased survival of cancer patients [1,2]. Both antitumor adaptive and innate cellular immunity are important for resistance of tumor growth and eventual elimination of cancer. Theoretically, antitumor cellular immune responses can be greatly enhanced by adoptive transfer of lymphocytes, a term encompassing a strategy in which autologous T or NK cells are acquired from a cancer patient and then activated and expanded *ex vivo* prior to reinfusion. Adoptive cell therapy of cancer, first demonstrated in mice more than 50 year ago [3], has gained momentum in recent years due to impressive clinical experiences with melanoma patients [4]. This approach is based on *ex vivo* expansion of large numbers of TILs and selection of tumor-specific T cell lines. The major effectors of TIL cells are phenotypically CD3^+^CD8^+^ T cells and their anti-tumor functions are MHC restricted [5]. In contrast to tumor antigen-specific immunotherapy, there is potential utility of non-antigen specific cell-based therapy. Many patients with cancer are ineligible for TIL-based therapy because their TILs do not expand sufficiently or because their tumors have lost expression of antigens or MHC molecules or have extremely low numbers of TILs. Cytokine-induced killer (CIK) cells are a heterogeneous population of effector CD8 T cells with diverse TCR specificities, possessing non-MHC-restricted cytolytic activities against tumor cells. Therefore, CIK cells can lyse tumor cells in a non-MHC-restricted manner and can serve as an alternative cellular immunotherapy. This review summarizes technical aspects of CIK, current clinical experiences and future clinical utility.

## The cellular characteristics of CIK

CIK cells are generated by *in vitro* expansion of peripheral blood lymphocytes (PBL) using anti-CD3 antibodies and IL-2. Short-term culture of human PBLs with IL-2 allows for proliferation and development of effector NK and nonspecific T-cells, with lymphokine-activated killer (LAK) activity [6,7]. LAK activity enables lysis of fresh tumor targets in a non-MHC restricted manner *in vitro* and also exerts *in vivo* anti-tumor effects. Nonetheless, using LAK cells as a tumor immunotherapy has not achieved much success clinically and is hampered by both the limited expansion of LAK cells *in vitro* and low cytolytic activity *in vivo*[8-10]. The human clinical extrapolation from murine immunotherapy models suggests that as many as 2 × 10^11^ human LAK cells may be necessary to allow adequate anti-tumor responses [11]. It is not practical to obtain such a large number of LAK cells for immunotherapy in humans. In addition, LAK-based therapy was limited by high toxicity due to the required *in vivo* infusion of IL-2. A solution for this problem was to induce more potent cytotoxic activities in harvested T cells. For this purpose, agonistic monoclonal antibodies (mAbs) against CD3 and IL-2 have been added to the PBMC culture. In such culture, more than 1000 fold expansion of cells can be achieved over 21-day *in vitro* culture. In addition, these cultured cells have potent cytolytic activity and can lyse tumor cells [11]. The lytic activity of these cells can be further increased by adding other cytokines such as IFN-γ and IL-1β [11]. The original culture conditions defining CIK activity was modified by adding IFN-γ 24 h before addition of anti-CD3 mAb and IL-2, and the term CIK cell was used to distinguish them from conventional IL-2 activated LAK cells [12]. With a substantial increase in cytotoxicity on a per cell basis and a higher proliferative response, CIK cells had a more than 70 fold increase in total cytolytic activity per culture when compared with standard IL-2-stimulated LAK cell activity [12].

Among expanded CIK cells, the cells with the greatest cytotoxicity against tumor cell lines express both the T-cell marker CD3 and the NK cell marker CD56. CD3^+^CD56^+^cells are rare in uncultured PBLs [13], consistent with the phenotype of resting naïve and memory T cells. When PBLs are cultured under CIK conditions for 21 days, more than 90% of the cells expanded are CD3^+^[14]. They are constituted by about 70% CD8^+^ and 30% CD4^+^ cells. The percentage of CD3^+^CD56^+^ cells also greatly increases and reaches a plateau after approximately 21 day of culture to more than 20 to 30% of the CIK cells, consistent with the generation of effector T cells in these cultures. Most of the CD3^+^CD56^+^ cells co-express CD2, TCRαβ, and CD8 but are negative for CD4, the helper T cell marker and CD16, an NK cell marker. The cytotoxicity mediated by CD3^+^CD56^+^ cells does not depend on MHC, similar to that mediated by NK cells. Interestingly, antitumor activity is restricted to the CD3^+^CD56^+^CIK and alloreactivity against HLA-mismatched PBMC is restricted to the CD3^+^CD56^−^ CIK [15]. These cells differ from NK cells because they do not mediate antibody-dependent cell-mediated cytotoxicity (ADCC). CD3^+^CD56^+^ cells expanded under these culture conditions are derived from CD3^+^CD56^-^T cells and not from CD3^-^CD56^+^NK cells [16]. Following culture, CD3^+^CD8^+^ but not CD3^+^CD4^+^ T cells express high levels of CD56. This is consistent with the fact that effector CD8 T cells possess high levels of cytotoxic activity [16]. In addition CIK cells are CD45RA^+^, CCR7, CD62L weakly positive, CD11a^+^, CD27^+^, CD28^+^, macrophage inflammatory protein 1a^+^, perforin^+^, Fas ligand^+^, suggesting these are largely terminally differentiated memory T cells [16]. Furthermore, the Vβ repertoire in CIK cells at the end of culture is of polyclonal pattern. Thus, the CIK culture condition generated a heterogeneous population of effector CD8 T cells with diverse TCR specificity but possess non-MHC-restricted cytolytic activities against tumor cells.

## Molecular triggers of CIK cytolytic activities

CIK cells possess potent cytotoxic activities against a number of tumor cell lines or freshly isolated tumor samples, including acute myeloid leukemia, chronic myeloid leukemia and B lymphoma cells [16-19]. Besides hematopoietic cancer cells [20], CIK cells exert potent *in vivo* antitumor effects on human solid tumors including liver cancer [21], gastric cancer [22], lung cancer [23], glioma [24] and others. In contrast, CIK cells demonstrate little or no cytolytic activity against normal bone marrow or spleen cells *in vitro*, with substantial specificity for tumor cells [24,25].

Although CIK cells are capable of undergoing degranulation upon stimulation with either agonistic anti-CD3 mAbs or susceptible target tumor cells, the molecules on malignant cells that stimulate cytolytic activities of CIK cells do not involve MHC-TCR interaction. Tumor cell-triggered cytotolytic activities in CIK can not be inhibited by neutralizing CD3 or HLA-class I mAbs. In addition, cyclosporin (CsA) and FK506 inhibit anti-CD3-mediated degranulation, but do not affect cytotoxicity of CIK cells against tumor target cells [26]. CIK cell binding to target cells and formation of cellular conjugates with tumor cells are required for CIK-mediated cytotoxicity against tumor cell lines [27]. Cell surface adhesion molecule leukocyte function associated antigen-1 (LFA-1) is known to participate in effector/target recognition and stable conjugate formation. CIK cells express LFA-1 and the most susceptible tumor cells express LFA-1 ligands such as ICAM-1, -2, and −3. CIK-tumor cell conjugate formation and cytotoxicity against tumor cells are strongly inhibited by anti–LFA-1 mAb, suggesting that LFA-1 has a critical role in binding to target as well as in enabling the cytotoxicity mediated by CIK cells [26,27]. Besides adhesion molecules, CIK cells express activating NK receptors, including NKG2D, DNAX accessory molecule-1 (DNAM-1), and low levels of NKp30. The NKG2D ligands such as MIC A/B and ULBPs are highly expressed on tumor cells [28]. Cell signaling through NKG2D, DNAM-1, or NKp30 results in CIK cell activation leading to degranulation and cytotoxicity. Neutralization of DNAM-1, NKG2D, and NKp30 by antibodies confirms that these molecules are involved in the TCR-independent tumor cell recognition and killing by CIK cells [26,27,29].

## Preclinical studies

The severe combined immunodeficiency (SCID) mouse model has been used for testing non-MHC-restricted antitumor effects with adoptive transfer of human CIK cells [12]. CIK cell infusion significantly prolongs survival of SCID mice injected with human lymphoma cells when compared with control animals injected with tumor cells alone or animals treated with LAK cells. Thus CIK cells seem to have superior *in vivo* antitumor activity in SCID mice, when compared with cells with LAK activity [16]. Using the SCID model, CIK cells have been shown to have *in vivo* antitumor effect against a number of hematopoietic and solid tumors [30].

Mouse CIK cells also possess anti-tumor cytolytic activities. When murine spleen cells are cultured under CIK culture conditions for 21 days, the majority of the cells are TCRαβ^+^ CD3^+^ CD8^+^ T cells [31]. Interestingly, approximately 20% to 50% of the cells express the NK markers NK1.1 and DX5, consistent with the phenotype of effector CD8 T cells. Cytotoxicity is greatest among cells expressing either NK1.1 or DX5. Expanded CD8^+^T cells produce high levels of T_H_1-type cytokines such as IFN-γ and inflammatory cytokines such as tumor necrosis factor α.

The availability of murine CIK cells allows testing their antitumor functions in immune-competent inbred mice. Thirty minutes following intravenous injection, CIK cells can be detected in the lungs, followed by distribution of the cells to other sites including the liver and spleen, within the next 16 hours. By 72 hours, significant numbers of transferred CIK cells are found in cells infiltrating tumor. CIK cells remain at the tumor site for more than 9 days. In addition, transferred mouse CIK cells demonstrate potent antitumor effect against both allogeneic and syngeneic transplanted tumor [32].

## Clinical studies

**Figure 1:**
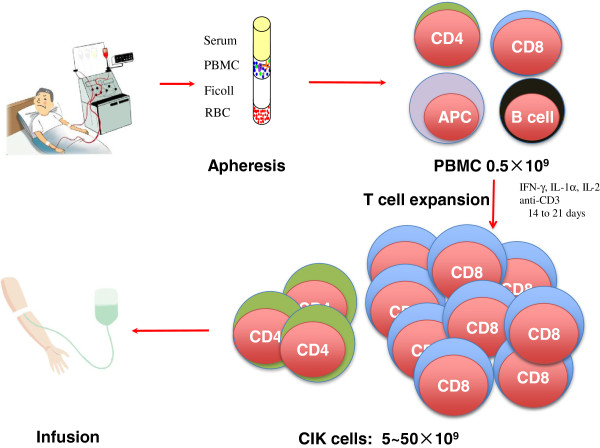
**Procedure for CIK preparation and infusion.** Peripheral blood mononuclear cells (PBMC) are isolated by apheresis. T cells are activated, expanded, and differentiated by anti-CD3 in the presence of cytokines including IFN-γ, IL-1α, and IL-2 for 14 to 21 days. These T cells, commonly called CIK, are then infused into patients.

CIK cells have been evaluated as an adoptive cell immunotherapy for cancer patients in a number of clinical trials (summarized in Table 1). Peripheral blood mononuclear cells (PBMC) were isolated by apheresis. T cells were then activated, expanded, and differentiated by anti-CD3 in the presence of cytokines including IFN-γ, IL-1α, and IL-2 for 14 to 21 days to generate CIK, which were subsequently infused into patients (Figure 1). In the first phase I clinical study, autologous CIK cells transduced with the interleukin-2 gene were infused in patients with metastatic renal cancer, colorectal cancer and lymphoma [33]. Transfected CIK cells could be detected for up to 2 weeks following infusion by analyzing expression of the IL-2 transgene. Six patients progressed, three patients were stable, and one patient with lymphoma developed a complete response. In three patients, WHO grade 2 fever was observed, and no major side-effect was observed despite the use of the IL-2 transgene in CIK cells.

**Table 1:** Published CIK cell clinical studies

**Year**	**Tumor type**	**No. of cases**	**Culture condition**	**Authors**
1999	Renal cell carcinoma Colorectal carcinoma Lymphoma	10	IFN-γ, anti-CD3, IL2 plus IL-2 transgene	Schmidt-Wolf IG et al. [33]
2005	Lymphoma	9	IFN-γ, anti-CD3, and IL2	Leemhuis T et al. [14]
2006	Gastric carcinoma	57	IFN-γ, anti-CD3, IL2 and IL-1alpha	Jiang JT et al. [34]
2007	Leukemia	11	IFN-γ, anti-CD3 and IL2	Introna M et al. [35]
2008	Non-small cell lung cancer	59	IFN-γ, anti-CD3, IL2 and IL-1alpha	Wu CP et al. [36]
2009	Hepatocellular carcinoma	127	IFN-γ, anti-CD3, IL2 and IL-1lalpha	Hui D et al. [37]
2009	Renal cell carcinoma and lymphoma	6	IFN-γ, anti-CD3, and IL2	Olioso P et al. [38]
2010	Gastric carcinoma	156	IFN-γ, anti-CD3, IL2, and IL-1alpha	Jiang JT et al. [39]
2011	relapsed hematologic malignancies	18	IFN-γ, anti-CD3, and IL2	Laport GG et al. [40]
2012	Renal cell carcinoma	74	IFN-γ, anti-CD3, IL2, IL-1beta	Liu L et al. [41]

CIK clinical studies were done on hematologic malignancies. A Phase I trial of autologous CIK cells for the treatment of patients with relapsed Hodgkin’s disease and non-Hodgkin’s lymphoma was carried out on nine patients with advanced Hodgkin’s disease (n=7) and non-Hodgkin’s lymphoma (n=2), who had relapsed after an autologous transplantation. Two patients had partial responses, and another two patients had stabilization of disease. Toxicity was minimal [14]. In another study, 6 advanced lymphomas were enrolled. One patient had CR with a median follow-up of 33 months [38]. Significant antitumor activity was reported against hematologic malignancies after hematopoietic cell transplant. A phase I study of allogeneic CIK cells in six leukemia patients relapsing after allogeneic haematopoietic stem cell transplantation (HSCT) was done. Acute GVHD (grade I and II) was observed in four patients. One patient had stable disease, one had hematologic improvement and three achieved complete responses [35]. The feasibility of CIK in patients with relapsed hematologic malignancies after allogeneic HCT was explored in another study. Eighteen patients were given CIK cell infusions at escalating doses. Acute GVHD grade I-II was seen in 2 patients, and 1 patient had limited chronic GVHD. Five patients had longer remissions. The therapy was well tolerated and induced a low incidence of GVHD [40]. These phase I clinical studies have yielded encouraging results and demonstrated the safety of using CIK cells as immunotherapeutic approach.

We (JJ and CW) reported the effects of autologous CIK cells together with chemotherapy on patients with advanced gastric cancer (stage IV) [34]. Fifty-seven patients were nonrandomly divided into two groups: those receiving chemotherapy plus CIK biotherapy and those treated with chemotherapy alone. Following CIK cell infusion, serum levels of tumor markers were significantly decreased, and the short-term curative effect and the quality of life (QOL) were improved in the patients treated by chemotherapy plus CIK cells when compared with patients treated by chemotherapy alone. Moreover, the 2-year survival was prolonged in the group treated by chemotherapy plus CIK cells when compared to the group treated with chemotherapy alone. Our follow-up study with a larger cohort of 156 patients further confirmed the benefit of CIK therapy [39]. In another report, we evaluated the clinical efficacy of chemotherapy when applied in combination with CIK cell therapy compared to chemotherapy alone [36]. Fifty-nine advanced non-small cell lung cancer (NSCLC) patients were randomly divided into two groups, chemotherapy alone (group A) and chemotherapy plus CIK cell transfusion (group B). Autologous CIK cells were induced from the patients’ peripheral blood mononuclear cells (PBMC) *in vitro*. We found that the QOL was improved in the patients treated by chemotherapy plus CIK biotherapy when compared with the patients treated by chemotherapy alone. The overall response rate (ORR) was 43.3% and 44.8% in groups A and B, respectively. The time to progression was 4.67 months in group A and 6.65 months in group B and the median survival time was 11.0 months in group A and 15.0 months in group B. Compared to patients in group A, the patients in group B had significantly longer progression-free survival (PFS) (p=0.042) and overall survival (OS) (p=0.029). No severe side-effect was observed in the CIK cell transfusion patients. These studies demonstrate that chemotherapy plus CIK cells has significant benefits for patients who suffer from advanced gastric cancer and lung cancer with no severe side-effects [34,36,39].

Two studies reported positive outcome for CIK therapy for patients with hepatocellular carcinoma. A clinical trial of postoperative adjuvant CIK immunotherapy following radical resection of hepatocellular carcinoma was reported. 127 patients were divided into 3 groups. After radical resection of the tumor, immunotherapy with CIK cells was performed for 3 courses in 41 patients (CIK-I group) and 6 courses in 43 patients (CIK-II group). The other 43 patients received no postoperative adjuvant therapy with CIK (the control group). The disease-free survival rates were significantly higher in the CIK-I group (p=0.001) and CIK-II group (p=0.004) than in the control group. In a separate study, adoptive CIK immunotherapy was also evaluated for reducing the recurrence of hepatocellular carcinoma (HCC) following minimally invasive therapy. 85 HCC patients after transcatheter arterial chemoembolization and radiofrequency ablation therapy were randomized to the immunotherapy group and the no adjuvant therapy group. The 1-year and 18-month recurrence rates of the CIK group were 8.9% and 15.6%, compared with 30.0% and 40.0% of the control group (both p values<0.05).

CIK has also been tried on renal cell carcinoma (RCC). The results of randomized study of autologous cytokine-induced killer cell immunotherapy in metastatic renal carcinoma have been reported recently [41]. 148 patients with metastatic clear cell RCC were randomized and assigned to two groups: i.e. autologous CIK cell immunotherapy (arm 1, n=74) and interleukin-2 treatment in combination with IFN-α-2a (arm 2, n= 74). The 3-year PFS in arm 1 was significant greater than that in arm 2 (18% versus 12%, p=0.031). And the 3-year OS in arm 1 was also significantly greater than that in arm 2 (61% versus 23%, p<0.001). The median PFS and OS in the CIK arm were significantly longer than those in the control arm (PFS, 12 *vs*. 8 months, p=0.024; OS, 46 *vs*. 19 months, p<0.001). In another study, 5 metastatic kidney carcinoma patients were enrolled. One patient had CR and two patients had SD with a median follow-up of 33 months [38]. The studies suggested that CIK cell immunotherapy could improve the prognosis of metastatic clear cell RCC with minor side effect.

Recently, the international registry on CIK Cells (IRCC) has been established to collect data worldwide and set standard criteria to report results from clinical trials performed with CIK cells [42]. 11 clinical trials with CIK cells were identified. Of the 384 patients, where clinical response information was available, 24 patients showed a complete response, 27 patients showed a partial response, 40 patients showed a minor response. The total response rate (RR) was 91/384 reported patients, 161 patients had a stable disease, 129 patients had progressive disease. Side effects of CIK cell treatment were minor. Disease-free survival rates were significantly higher in patients treated with CIK cells than in a control group without the CIK treatment.

We have recently reviewed the results published in Chinese in a separate paper [43]. We collected results from 24 clinical trials. Among these trials, 936 patients were treated with CIK cells, including 525 men and 246 women. In five studies, CIK cells were co-cultured with dendritic cells (DCs). The total number of CIK cells used ranged from 6×10^6^ to 1.5×10^10^. Of the 563 patients, where a clinical response was reported, 40 had CR, 126 had PR, 125 had MR, 135 had SD, 58 had PD. The total response rate (RR) was 51.7% (291/563). The toxicities were minimal. These results demonstrate that CIK cell treatment is a promising and safe modality for treating solid tumor.

## Improving CIK therapy for epithelial cancer

The total antitumor cytolytic activity within cell culture has been used as the sole marker for the success of CIK generation [11,12]. This is influenced by T cell proliferation as well as the development of effector function. Most of the studies use T cells cultured for 14 to 21 days. Cytokines such as IFN-γ, IL-1, IL-2, IL-7, IL-15, and IL-12 have been evaluated for their ability to promote the CIK generation [12,44-48]. According to the standard CIK culture condition, IFN-γ is added at the beginning of the culture and 24 h before the addition of anti-CD3 mAb and IL-2 [12]. This is likely due to its role in promoting IL-12 production by antigen presenting cells as well as its ability to promote generation of autophagy and antigen cross-presentation. In contrast, IL-2, IL-7, IL-15 and IL-12 can directly promote T cell proliferation/survival and development of cytolytic effector functions. Other pro-proliferative cytokines such as IL-21 and IL-23 need to be tested separately or in combination with the cytokines currently used in the CIK culture to examine whether they can further promote CD8 T cell proliferation. In addition to IL-1, other members of the IL-1 protein family such as IL-18 and IL-33 promote CD8 and Th1 effector functions [49,50]. These cytokines should be tested in CIK cultures to determine whether they can promote cytolytic activities of CIK cells. Furthermore, co-stimulatory molecules such as CD28 and 4-1BB may also promote the efficient production of CIK cells.

The existence of anti-tumor-specific T-cells in cancer patients has been well documented. A potential mechanism of action for CIK is enhancement of a recall antitumor response by CD45RO^+^ memory T-cells. It is possible that spontaneous tumor-specific T cells are expanded with other non-specific T cells during CIK culture. Study on CMV-specific T cells showed that the percentage of CMV tetramer^+^ T cells did not significantly change from the beginning to the end of the culture, suggesting that CMV tetramer^+^ T cells expanded with similar efficiency with the total CD8 T cells [27]. Thus, tumor-specific components of CIK cells might be responsible for the clinical benefit observed in some cancer patients [51].

Besides spontaneous antitumor cellular responses, some chemotherapy regimens can induce tumor-specific T cells in cancer patients by inducing the immunogenic death of tumor cells or by engaging immune effector mechanisms [52]. Consistent with this idea, our previous studies showed that CIK following chemotherapy significantly prolonged the survival of patients with advanced gastric cancer [34,36,39]. The underlying mechanism needs to be further studied in order to improve the combined therapy. Future studies should focus on determining which chemotherapy regimen has the greatest synergy with CIK and the associated mechanisms. A recent study showed that autophagy is required for immunogenicity of tumor under chemotherapy. This is due to the fact that suppression of autophagy inhibits the release of adenosine triphosphate (ATP), a key danger signal, from dying tumor cells [53,54]. Thus, thermotherapy that enhances autophagy in cancer cells might synergize with CIK therapy.

CIK cells can be rendered tumor antigen-specific by transduction with the retrovirus vectors encoding either high affinity T cell receptors (TCR) or chimeric antibody-T cell receptors (CAR) which recognize tumor antigens [55]. Patients with melanoma have been treated with T cells engineered to express high affinity TCRs specific for melanoma antigens MART-1, gp100 and NY-ESO-1 [56-58]. Objective clinical responses have been reported, although some patients experienced autoimmune responses, suggesting TCRs need to be carefully selected [56,57]. CARs consist of the variable region of a tumor specific monoclonal antibody fused to an intracellular domains CD3 zeta and CD28. Autologous activated T cells genetically engineered to express the anti-CD19 CAR have been effective in treating patients with lymphoma and chronic lymphocytic leukemia (CLL) [59-62]. The side effect of this approach includes elimination of normal B cells in these patients. Besides the genetic engineering approach, tumor targeting can also be made specific using a bi-specific antibody with one part recognizing a surface tumor antigen and the other part binding to a T cell membrane protein [63-65]. The clinical efficacy of such approach remains to be evaluated.

It was originally reported that CIK cells could be *ex vivo* expanded up to 1000 fold with standardized conditions. However, it is a practical clinical problem that individual variability exists in expansion rates. This is likely due to immune suppression in cancer patients. Myeloid derived suppressor cells contaminated in the PBMC preparation or defective antigen presenting cells might inhibit T cell expansion [66]. In addition, T cells in cancer patients might be in a stage of anergy and exhaustion [67]. Many new approaches should be tested including using autologous dendritic cells (DC-CIK) or allo-antigen presenting cells [68].

Due to their abilities to migrate to tumor site, CIK cells can also be used to deliver bio-therapeutic agents to the tumor site. One study combined CIK cells with an oncolytic viral therapy and achieved directed delivery to, and regression of, tumors in mouse models [69]. The CIK cells retained their ability to traffic to and to infiltrate the tumor effectively before releasing the virus and the modified vaccinia virus remains inactive until the CIK cell encounters with the tumor. These results highlight the idea that CIK cells can be used as a vehicle for delivering biologic drugs directly to the tumor microenvironment. In addition, finding ways to limit induction of autophagy and resistance to immune-mediated effectors are also promising strategies to couple with adoptive transfer of CIK cells [70-72]. Trafficking of CIK cells is hard to investigate in cancer patients. One approach to further study the trafficking is to use various humanized mouse models [73]. *In vivo* trafficking can also be studied by tracking genetically engineered T cells upon infusion [74].

The survival of adoptively transferred CIK cells needs to be further studied in patients and humanized mouse models. CIK cells are composed of a mixture of effector and central memory T cells. Effector T cells, although possess potent immediate cytotoxic activities, produce a large amount of IFN-γ and express high levels of NKG2D, are known to be prone to apoptosis. Central memory T cells and memory stem T cells, although require restimulation *in vivo* to gain effector functions, are more resistant to apoptosis than effector T cells [75-78]. Therefore, studying the in vivo survival of distinctive subsets of CIK cells and its relationship to clinical outcome should help improve this therapy.

So far there is no reliable biomarker for estimating clinical responses and predicting outcomes of CIK therapy. Among many changes in the blood, it has been shown that CD4/CD8 ratio and percentage of NK cells are significantly increased in patients infused with CIK [34,36]. Whether this or other markers correlate with clinical outcome is not known [34,36]. The frequency of tumor antigen-specific T cells should be studied in future clinical trials to examine whether it is associated with better patient outcome. In addition to blood marker, immunosuppressive molecules that are expressed in the tumor microenvironment, such as members of B7 family, should be further studied to determine whether they can serve as prognostic markers for the CIK therapy [79,80].

## Conclusions

Current clinical studies suggest that CIK is a promising and safe modality for treating malignancies. Multicenter clinical trials are warranted to further establish the validity of this therapeutic approach and optimize the CIK treatment protocol. This therapy should be further improved through increasing the specificity of CIK cells via immunological and genetic engineering approaches and identifying robust predictive biomarkers for patient stratification. In addition, we believe combining chemotherapy, radiotherapy or other immunotherapy approaches with CIK will further improve cancer therapy and prolong survival of cancer patients.

## Competing interests

The authors declare that they have no competing interests.

## Authors’ contributions

JJ: drafted the manuscript. CW: drafted parts of the manuscript. BL: drafted the manuscript. All authors read and approved the final manuscript.
